# Hemoglobin levels and frailty status in generally healthy and active community-dwelling adults age 70 years and older in the three-year DO-HEALTH study

**DOI:** 10.1007/s00277-025-06558-w

**Published:** 2025-09-08

**Authors:** Simon Broghammer, Michael Gagesch, Maud Wieczorek, Reto W. Kressig, Markus G. Manz, E. John Orav, Heike A. Bischoff-Ferrari

**Affiliations:** 1 Department of Medical Oncology and Hematology, Comprehensive Cancer Center Zurich, University Hospital Zurich and University of Zurich, University of Zurich, Zurich, Switzerland; 2 Centre on Aging and Mobility, University of Zurich, Zurich, Switzerland; 3 Department of Geriatrics and Aging Research, University of Zurich, Zurich, Switzerland; 4 University Clinic for Aging Medicine, City Hospital Zurich Waid, Zurich, Switzerland; 5 Department of Geriatrics, University of Basel, Basel, Switzerland; 6 University of Basel Department of Aging Medicine, Felix Platter Basel, Burgfelderstrasse 101, Basel, 4055 Switzerland; 7 Swiss Healthy Longevity Campus, University of Basel, Basel, Switzerland; 8 Department of Biostatistics, Harvard T.H. Chan School of Public Health, Boston, MA USA

**Keywords:** Frailty risk factors, Multicenter clinical trial, Healthy aging, Geriatrics and hematology

## Abstract

While frailty and anemia are prevalent conditions in aging linked to adverse outcomes, their relationship remains understudied in generally healthy older adults. We conducted a post-hoc observational study among all participants of DO-HEALTH, the largest European clinical trial designed to support healthy aging. Our analysis examined whether baseline hemoglobin levels and anemia are associated with being at least pre-frail at baseline and any yearly follow-up time point over three years. Frailty status was assessed by the Fried frailty phenotype at baseline and at each annual follow-up visit. Participants were considered at least pre-frail by the presence of any frailty domain (fatigue, unintentional weight loss, slowness, weakness, low activity). For both hemoglobin levels and anemia as predictors, we used regression models based on generalized estimating equations controlling for potential confounders. In total, 2,123 of 2,157 participants had available frailty status and hemoglobin levels at baseline and were included in the analysis (mean age 74.9 years; 61.7% women). At baseline, 46.4% (*n* = 986) were at least pre-frail, while the mean hemoglobin level was 139.7 g/L (SD 12.5), and 6.7% (*n* = 141) had anemia. In our multivariate prospective analysis, each 10-g/L decrease in baseline hemoglobin levels was associated with 8% higher odds of pre-frailty (adjusted Odds Ratio [aOR] 1.08, 95%CI 1.01–1.16, *p* 0.02), and baseline anemia was associated with 39% higher odds of pre-frailty over three years (aOR = 1.39, 95%CI 1.02–1.88, *p* 0.03). In conclusion, our results provide novel insights into the association between hemoglobin levels, anemia, and pre-frailty in generally healthy and active older adults.

## Introduction

Frailty in older adults has been commonly defined by a physical frailty phenotype, which arises from multi-system dysregulation and is characterized by a reduction in functional capacity. Thus, frailty is associated with increased vulnerability to external stressors [1]. Frailty is most frequently observed in the rapidly growing population of older adults and constitutes a considerable burden at both the individual and public health levels [2]. In Europe, the overall prevalence of frailty in community-dwelling adults aged 50 years and older is estimated at 7.7%, with an additional 42.9% at increased risk of becoming frail (i.e. being pre-frail) [3]. Even more, frailty prevalence is considerably higher at older age, reaching 33% in the age group 85 years and older [3].

According to Fried et al. [4], five components (fatigue, unintentional weight loss, slowness, weakness, and low activity level) constitute physical frailty, with the presence of one or two representing pre-frailty and three or more representing frailty [5]. Pre-frail individuals may compensate better for external disturbances of homeostatic mechanisms compared to those affected by frailty. However, pre-frailty is still associated with increased risks of mortality, falls, disability, hospitalization rates [4], cardiovascular diseases [6, 7] and multimorbidity [8] in community-dwelling older adults.

Some interventions, including physical exercise and nutritional supplementation, have proved effective in preventing or delaying the advancement of pre-frailty and frailty [9]. However, preventing and managing pre-frailty and frailty still poses a major challenge in clinical practice [10]. Therefore, early identification of at-risk individuals on a frailty trajectory and the investigation of potentially modifiable risk factors might serve as a valuable strategy towards the promotion of healthy and active aging [2].

Among such risk factors, the relevance of anemia and low hemoglobin levels as key indicators of various clinical conditions in older adults has been extensively explored [11, 12]. The World Health Organization reference values for hemoglobin levels [13] are most often used for defining anemia. By this definition, anemia affects approximately 12% of community-dwelling older adults in high-income countries [14]. Anemia in older age has been identified to be independently associated with increased mortality [15] but also with reduced mobility and reduced muscle strength [16, 17], characteristic features also in the development and the progression of frailty [4]. However, the appropriateness of these reference ranges in the older adult population has been disputed [18] and a recent WHO guideline [19] did not specify a cutoff point for anemia in adults age over 65 years.

So far, scientific evidence on the association of anemia and frailty status in community-dwelling older adults is largely based on cross-sectional studies. In 2018, a meta-analysis including 17 cross-sectional studies with low to moderate risk of bias reported a two-fold increased odds of being frail among anemic individuals, compared to their non-anemic counterparts [20]. To date, only three longitudinal cohort studies investigated the association between hemoglobin levels and the development of frailty in community-dwelling older adults, reporting conflicting results [21–23]. Among those, a population-based study in 1,666 Australian men aged 70 years and older found a significant increase of 80% in the odds of becoming frail over five years of follow-up in anemic participants [21]. Conversely, another population-based study reported that anemia was associated with a 17% reduction in the odds of becoming pre-frail or frail and a 16% reduction in the odds of become frail when being pre-frail among 2,925 male and female Italian adults age 65 and older with a mean follow-up of 4.4 years [22]. Lastly, a smaller longitudinal study among 254 older adults from the UK did not find a significant association between baseline hemoglobin levels and incident frailty over 10 years of follow-up [23].

Given the current limited scientific evidence on the association between hemoglobin levels and frailty status in European male and female older adults, results from a large, high-quality, multicenter, longitudinal study in generally healthy, community-dwelling older adults appears valuable. The present study aimed to investigate the association of hemoglobin levels and prevalent anemia at baseline with the odds of being at least pre-frail at baseline and at any yearly follow-up time point over three years in generally healthy and community-dwelling adults, age 70 years and older from five European countries.

## Methods

### Study design and participants

This study is a post-hoc observational analysis of the DO-HEALTH study, a multi-center, double-blind, randomized controlled clinical trial designed to support healthy aging in community-dwelling European older adults. The trial examined the individual and combined effects of omega-3 fatty acids and/or vitamin D supplementation, and a simple home exercise program, each compared to placebo or control exercise over three years of follow-up [24]. In total, 2,157 participants aged 70 years and older were recruited at seven centers from five European countries: Zurich, Basel, Geneva (Switzerland), Berlin (Germany), Innsbruck (Austria), Toulouse (France) and Coimbra (Portugal). Inclusion criteria were the absence of major health events in the five years prior to enrollment (including a diagnosis of cancer and cardiovascular diseases), sufficient mobility, and good cognitive function. For the present study, participants with missing information for hemoglobin levels and frailty status at baseline were excluded from the analysis. All participants provided written informed consent prior to study enrollment. Further details on DO-HEALTH have been reported earlier [25].

### Outcomes

As our primary outcome, frailty status (non-frail, pre-frail, frail) was assessed at baseline and annually over the three years of follow-up. We defined frailty according to the Fried physical frailty phenotype [4], with limited adaptions to its five domains (weakness, fatigue, unintentional weight loss, low gait speed, and low activity level) in order to match the available variables from the DO-HEALTH dataset [26]. For weakness, grip strength was measured in Kilopascal (kPa) from the best of three consecutive trials at the dominant hand using a Martin Vigorimeter (KLS Martin Group, Tuttlingen, Germany). We used cut-points by the lowest quintile approach as done by Fried et al., stratified by sex and age group (< 75 years and ≥ 75 years) [27]. Fatigue was operationalized by a positive answer to the self-reported question: “In the last month, have you had too little energy to do things you wanted to?” from the SHARE questionnaire [28]. Involuntary relevant weight loss was defined as > 4.5 kg in the last year, reported at the baseline visit or > 5% in one year, as measured at a follow-up visit. Low gait speed was defined as ≤ 0.65m/s (for men ≤ 173 cm and women ≤ 159 cm in height), and ≤ 0.76 m/s (for men > 173 cm and women > 159 cm in height) and obtained from a 4-meter walking test as part of the Short Physical Performance Battery. Low activity level was defined as an answer of “Less than once a week” to the self-report question: “How often do you engage in activities that require a low or moderate level of energy such as gardening, cleaning the car, or doing a walk?” from the SHARE questionnaire. At baseline and each follow-up time point, participants were classified as non-frail (0 positive items) or at least pre-frail if they scored positive in at least one of the five Fried phenotype domains, resulting in up to four outcome measures per participant.

### Exposures

Blood samples were collected after an overnight fast by medically trained staff. Hemoglobin concentration, expressed in g/L, was measured in the whole blood during the baseline visit and measured with standard automated and routine diagnostic lab quality-controlled analyzers available at the local analysis facility of each study center. Both baseline hemoglobin levels and anemia, defined as hemoglobin levels less than 130 g/L for men and less than 120 g/L for women, according to the World Health Organization guidelines [13], were considered as exposures.

### Covariates

Participants’ sociodemographic and clinical characteristics including age, sex, body mass index (BMI) and history of fall in the 12 months preceding randomization were recorded at baseline. The baseline number of comorbidities was assessed with the Self-Administered Comorbidity Questionnaire by Sangha et al. [29]. Baseline iron deficiency was defined as soluble Transferrin Receptor levels ≥ 28.1 nmol/L [30–32], measured by Tina-quant Transferrin ver.2 Test on a cobas c 502 analyser (Roche, Switzerland) using an Immunoturbidimetric assay [32]. Kidney function was assessed with creatinine levels and estimated glomerular filtration rate (eGFR, ml/min/1.73 m²) using the Cockcroft-Gault formula [33], at baseline and over follow-up. Vitamin B12 levels were measured on a cobas e602 analyser (Roche, Switzerland) using an electrochemiluminescence assay at baseline and over the follow-up.

### Statistical analysis

Baseline demographic and clinical characteristics of the study population are presented overall and for non-frail and at least pre-frail participants at baseline, separately. Differences between groups were tested using the Wilcoxon rank sum test, t-test or chi-square test, for non-normal, normal and categorical variables, respectively.

We used regression models based on generalized estimating equations to investigate the relationship between a 10 g/L decrease in baseline hemoglobin levels and baseline anemia with regard to the odds of being at least pre-frail at baseline and at each yearly follow-up time point (resulting in up to four outcome measures per participant). An unstructured covariance matrix was specified to account for within-subject correlation due to repeated measures. The existence of a non-linear relationship between hemoglobin levels and being at least pre-frail at baseline and at each yearly follow-up time point was tested by checking the significance of quadratic and cubic terms of hemoglobin in the minimally- and fully-adjusted models. Minimally-adjusted models controlled for DO-HEALTH randomization stratification factors including study center, treatment allocation, age, sex, and prior fall, as well as baseline BMI. Fully-adjusted models additionally included baseline number of comorbidities, as well as vitamin B12 levels and eGFR at baseline and over the follow-up. All results were expressed as odds ratios (OR) along with their respective 95% confidence intervals (CI). We planned to conduct subgroup analyses by sex, age group (70–74 or 75+) and baseline iron status in case of indication for significant effect modification by checking the significance of the respective interaction terms between subgroup factors and baseline hemoglobin levels or baseline anemia status.

Given the potential influence of anticoagulants or platelet aggregation inhibitors on hemoglobin levels, we excluded participants who reported the use of these drugs over the follow-up in a sensitivity analysis. In a second sensitivity analysis, we excluded participants who were at least pre-frail at baseline to explore whether baseline hemoglobin levels and anemia were associated with the development of at least pre-frailty over time (resulting in up to three outcome measures per participant).

Statistical analyses involved using SAS version 9.4 (SAS Institute, Cary, NC). Two-sided p-values < 0.05 were considered statistically significant.

## Results

### Baseline characteristics of the study population

Of the 2,157 trial participants, 34 (1.6%) had missing frailty status at baseline and 2 (0.1%) had missing hemoglobin levels at baseline. In all, 2,123 (98.4%) participants were included in the analyses. Baseline characteristics are presented in Table 1. Overall, the mean age was 74.9 years (SD 4.5) and 61.7% of participants were women. At baseline, 1,137 (53.6%) and 986 (46.4%) individuals were non-frail and at least pre-frail, respectively. The mean hemoglobin levels at baseline were 139.7 g/L (SD 12.5) with an overall prevalence of anemia of 6.7%. Compared to their non-frail counterparts, individuals who were at least pre-frail at baseline were more likely to be older (75.5 vs. 74.3 years, *p* < 0.001), to be women (67.3 vs. 56.4%, *p* < 0.001), to have a higher mean BMI (26.6 vs. 26.1 kg/m^2^, *p* = 0.01), to have higher vitamin B12 levels (389 vs. 374 ng/L, *p =* 0.01), to have a higher number of comorbidities (2.1 vs. 1.4%, *p* < 0.001), to have a higher prevalence of prior falls (44.9 vs. 38.6%, *p =* 0.003), and to be iron-deficient (29.5 vs. 24.2%, *p* = 0.006). The prevalence of at least pre-frailty at each time point over the follow-up among non-frail participants at baseline are presented in Table 2.

**Table 1:** Baseline characteristics of the study population – overall and by frailty status at baseline

	Overall	Non-frail	At least pre-frail	*P*-value ^a^
	*N* = 2,123	*N* = 1,137 (53.6%)	*N* = 986 (46.4%)	
Age, years, mean (SD)	74.9 (4.5)	74.3 (4.0)	75.5 (4.8)	< 0.001
Women, N (%)	1,331 (61.7)	642 (56.4)	664 (67.3)	< 0.001
Body mass index, kg/m², mean (SD)	26.3 (4.3)	26.1 (4.0)	26.6 (4.5)	0.01
Prior fall, N (%)	903 (41.9)	439 (38.6)	443 (44.9)	0.003
No of comorbidities, mean (SD)^b^	1.7 (1.4)	1.4 (1.2)	2.1 (1.6)	< 0.001
eGFR, ml/min/1.73 m², mean (SD)	62.8 (10.3)	63.0 (9.8)	62.7 (10.9)	0.43
Vitamin B12 levels, ng/L, median (IQR)	380 (295–493)	374 (292–479)	389.0 (299–509)	0.01
Iron deficiency, N (%)^c^	573 (26.8)	274 (24.2)	288 (29.5)	0.006
Hemoglobin levels, g/L, mean (SD)	139.7 (12.5)	141.4 (12.1)	137.8 (12.6)	< 0.001
Anemia, N (%)				
Yes	141 (6.7)	54 (4.8)	87 (8.8)	< 0.001
No	1980 (93.4)	1083 (95.2)	897 (91.2)	

eGFR: estimated glomerular filtration rate

^a^ Differences between non-frail and at least pre-frail participants at baseline were tested using the Wilcoxon rank sum test, t-test or chi-square test, for non-normal, normal and categorical variables, respectively

^b^ Self-reported number of comorbidities was assessed by the Sangha questionnaire, range 0–13

^c^ Iron deficiency was defined by soluble transferrin receptor levels ≥ 28.1 nmol/L

**Table 2:** Frailty status at each time point among participants who were non-frail at baseline (*n* = 1,137)

	Year 1	Year 2	Year 3
Non-frail	668 (63.2%)	564 (55.9%)	514 (51.1%)
At least pre-frail	389 (36.8%)	445 (44.1%)	492 (48.9%)

### Association between baseline hemoglobin levels and frailty status at baseline and at any yearly follow-up time point

We found a linear relationship between baseline hemoglobin levels and the odds of being at least pre-frail at baseline and at any yearly follow-up time point over three years. After controlling for treatment allocation, study center, age, sex, prior fall and BMI, each 10-g/L decrease in baseline hemoglobin levels was statistically significant and linearly associated with 11% higher odds of being at least pre-frail (OR 1.11; 95% CI: 1.04–1.19; *p* < 0.001). When additionally controlling for number of comorbidities, vitamin B12 levels and kidney function, this association remained significant with 8% higher odds of being at least pre-frail with each 10-g/L decrease in baseline hemoglobin levels (OR 1.08; 95% CI 1.01–1.16; *p* = 0.02) (Table 3).

**Table 3:** Association between hemoglobin levels and anemia at baseline and the odds of being at least pre-frail at baseline and over three years

	Minimally adjusted model^a^		Fully adjusted model^b^	
	Odds ratio (95% CI)	P-value	Odds ratio (95% CI)	P-value
Hemoglobin levels, (g/L)	**1.11** (1.04, 1.19)^c^	< 0.001	**1.08** (1.01, 1.16)^c^	0.02
Anemia (vs. no anemia)	**1.47** (1.10, 1.98)	0.01	**1.39** (1.02, 1.88)	0.03

^a^ The models control for treatment allocation, study center, age, sex, prior fall, baseline body mass index

^b^ Additional adjustment on baseline number of comorbidities, vitamin B12 levels (time-varying covariate), and estimated glomerular filtration rate (time-varying covariate)

^c^ Odds ratio per 10-g/L decrease in baseline hemoglobin levels (g/L)

### Association between baseline anemia and frailty status at baseline and at any yearly follow-up time point

Overall, we found participants with prevalent anemia at baseline had 47% higher odds of being at least pre-frail at baseline and at any yearly follow-up time point over three years when compared to their non-anemic counterparts (OR 1.47; 95% CI 1.10–1.98; *p* = 0.01). Results were consistent when further adjusting the model on the number of comorbidities, vitamin B12 levels and kidney function, with 39% higher odds of being at least pre-frail at baseline and at any yearly follow-up time point over three years among participants with prevalent anemia at baseline (OR 1.39; 95% CI 1.02–1.88; *p* = 0.03) (Table 3).

### Subgroup analysis

There was no indication of a significant effect modification by sex, age or baseline iron status in the association between baseline hemoglobin levels and being at least pre-frail at baseline and at any yearly follow-up time point over three years in the fully adjusted models. As a result, we did not proceed with the originally planned subgroup analysis. Findings were similar when considering baseline anemia as exposure (Table 4).

**Table 4:** Effect modification by subgroup

Exposures	Subgroup variable	*P*-values for interaction between subgroup and exposure
Hemoglobin levels	Sex	0.92
	Age	0.96
	Baseline iron status	0.96
Anemia	Sex	0.80
	Age	0.12
	Baseline iron status	0.96

Results from separate regression models based on generalized estimating equations controlling for treatment allocation, study center, age, sex, prior fall, baseline body mass index, baseline number of comorbidities, vitamin B12 levels (time-varying covariate), and estimated glomerular filtration rate (time-varying covariate)

### Sensitivity analyses

When excluding 738 participants who reported the use of anticoagulants or platelet aggregation inhibitors over the follow-up, the directions of associations observed in the main analysis remained overall consistent. However, most of the associations lost statistical significance in minimally- and fully adjusted models for both exposures (Table 5). In our second sensitivity analysis, which excluded 986 participants who were at least pre-frail at baseline, the directions of the associations again remained consistent with the main analysis, though they did not reach statistical significance (Table 6).

**Table 5:** Association between baseline hemoglobin levels, baseline anemia and being at least pre-frail at baseline and over three years – Sensitivity analysis excluding participants who reported the use of anticoagulants or platelet aggregation inhibitors over three years (*n* = 1,385)

	Minimally adjusted model^a^		Fully adjusted model^b^	
	Odds ratio (95% CI)	p-value	Odds ratio (95% CI)	p-value
Hemoglobin levels (g/L)	**1.10** (1.01, 1.19)^**c**^	0.04	1.06 (0.98, 1.16)^c^	0.16
Anemia (vs. no anemia)	1.31 (0.88, 1.95)	0.18	1.26 (0.83, 1.92)	0.27

^a^ The models control for treatment allocation, study center, age, sex, prior fall, baseline body mass index

^b^ Additional adjustment on baseline number of comorbidities, vitamin B12 levels (time-varying covariate), and estimated glomerular filtration rate (time-varying covariate)

^c^ Odds ratio per 10-g/L decrease in baseline hemoglobin levels (g/L)

**Table 6:** Association between baseline hemoglobin levels, baseline anemia and being at least pre-frail at baseline and over three years – Sensitivity analysis excluding participants who were at least pre-frail at baseline (*n* = 1,137)

	Minimally adjusted model^a^		Fully adjusted model^b^	
	Odds ratio (95% CI)	p-value	Odds ratio (95% CI)	p-value
Hemoglobin levels (g/L)	1.05 (0.95, 1.15)^**c**^	0.31	1.04 (0.95, 1.15)^c^	0.37
Anemia (vs. no anemia)	1.38 (0.92, 2.05)	0.12	1.38 (0.93, 2.07)	0.11

^a^ The models control for treatment allocation, study center, age, sex, prior fall, baseline body mass index

^b^ Additional adjustment on baseline number of comorbidities, vitamin B12 levels (time-varying covariate), and estimated glomerular filtration rate (time-varying covariate)

^c^ Odds ratio per 10-g/L decrease in baseline hemoglobin levels (g/L)

## Discussion

In this prospective observational study among 2,123 generally healthy and community-dwelling adults enrolled in a clinical trial from five European countries, we found that lower hemoglobin levels, as well as prevalent anemia at baseline, were significantly associated with higher odds of being at least pre-frail at baseline and at any yearly follow-up time point over three years. These associations remained significant even after controlling for several potential confounders.

When excluding participants who were at least pre-frail at baseline, the observed associations lost statistical significance but remained directionally consistent with the main analysis. This attenuation may likely be due not only to the reduced sample size and statistical power, but also to the creation of a healthier analytical sample that may have been less vulnerable to developing frailty, thereby weakening the observed association between hemoglobin and pre-frailty. Similarly, in the sensitivity analysis excluding participants who reported the use of anticoagulants or platelet aggregation inhibitors during follow-up, the associations lost statistical significance. This may reflect the removal of individuals with underlying cardiovascular conditions that are associated with lower hemoglobin levels and increased frailty risk, and potentially also the removal of individuals with subclinical blood loss associated with the use of anticoagulant drugs. Excluding these participants may have reduced residual confounding by indication, but also limited the variability required to detect a significant association.

To our knowledge, this is the first study prospectively investigating the association of hemoglobin levels and anemia with frailty status in generally healthy European community-dwelling older adults enrolled in a multi-national study. Our findings appear to be in line with a prior Australian longitudinal study which reported a 80% increased odds of prevalent frailty (OR 2.90; 95% CI 1.87–4.81; *p* < 0.0001) and incident frailty (OR 1.80; 95% CI 1.14–2.85; *p* = 0.01) over a five-year follow-up in individuals with anemia at baseline [21]. Nonetheless, the study population was exclusively male, which limits the generalizability and comparability to our findings. In contrast, an earlier Italian population-based study in 2,925 participants aged 65 and older reported lower odds of progression from non-frail to pre-frail/frail (OR 0.83; 95% CI 0.72–0.96; *p* < 0.05) or from pre-frail to frail over a follow-up of 4.4 years in individuals with anemia (OR 0.84; 95% CI 0.72–0.99; *p* < 0.05) [22]. However, the frequency of the outcome assessment and definitions of the frailty domains fatigue, unintentional weight loss and low activity level differed in between studies and direct comparisons are therefore limited. In addition, the present study treated frailty status as a binary outcome at each time point and was not designed to model frailty transitions or reversibility, which further limits comparability. Also, in the above-mentioned Italian study [22], participants with anemia were shown to have a 72% increase in mortality. It may therefore be likely that the progression of frailty occurring between the two evaluation periods in these individuals was not fully captured in the final frailty assessment due to their potential premature mortality.

Several pathophysiological mechanisms may explain the manifestation and the progression of frailty among older adults with anemia. In animal models, anemia has been shown to diminish oxygenation of muscle tissue and lead to impaired muscle performance [34]. Thus, anemia might contribute to low muscle mass, impaired exercise tolerance and reduced physical activity, which in turn may promote the development of frailty [4]. Recent research also emphasized the associations between an age-dependent pro-inflammatory state and both anemia and frailty [12]. However, the pathophysiological pathways that link the two conditions remain largely hypothetical to date. Our assessment of potential modifiers in this relationship, including age, sex, and baseline iron deficiency did not reveal any effect modification, suggesting the association between either hemoglobin levels or anemia and frailty status exists independently and might be driven by the syndromic character of frailty.

Our findings on the association between lower hemoglobin levels and higher odds of being at least pre-frail may have relevant implications for clinical practice. Hemoglobin concentration assessment, being both cost-effective and easily administrable across a range of clinical environments, has the potential to serve as a valuable marker in the context of pre-frailty and progression to frailty among older adults. Furthermore, the assessment of anemia and hemoglobin levels may complement the use of frailty screening tools as widely available parameters within primary care settings [35], providing a more comprehensive understanding of older patients’ risk profiles.

To the best of our knowledge, no intervention studies to date have been specifically designed to test whether correction of anemia or improvement in hemoglobin levels can prevent the onset or progression of frailty. Previous longitudinal research supports the notion that higher hemoglobin levels may play a role in frailty improvement. For example, He et al., in a prospective cohort of 1,433 community-dwelling older adults in China followed over two years, found that higher hemoglobin levels were significantly associated with the likelihood of transitioning from frailty to a non-frail state [36]. However, He et al. assessed frailty using a principal component analysis–based Frailty Index, a concept of accumulating deficits that partially overlaps with, but is also distinct from, the Fried frailty phenotype used in our study. These findings appear promising but require confirmation in future clinical trials and prospective studies specifically designed to assess the impact of hemoglobin improvement on frailty prevention or reversal using standardized frailty criteria.

The present study has several strengths. First, we utilized data from DO-HEALTH, the largest European clinical trial on healthy aging. Since DO-HEALTH was a randomized controlled trial, all procedures followed strict protocols and participants were meticulously phenotyped, with the collection of a wide range of demographic, clinical and laboratory data at baseline and at each annual clinical visit over three years. Consequently, we were able to strengthen the validity of our findings in adjusting on a wide range of clinically relevant potential confounders. Second, frailty, which was a secondary endpoint in the DO-HEALTH trial, was assessed in a standardized way following a widely accepted definition (Fried physical frailty phenotype) at baseline, and annually over the duration of follow-up.

Some limitations of our study must be considered. First, we report on a secondary analysis of the DO-HEALTH study. Thus, our study sample comprises of generally healthy, community-dwelling older adults from five European countries, enrolled in a clinical trial. Although DO-HEALTH aimed to target a potentially pre-frail population by including at least 40% of individuals who had reported a fall in the prior year to enrollment, the prevalence of anemia was lower than expected in the general population [14]. Therefore, our findings may not be generalizable to more vulnerable older population groups. Second, since hemoglobin levels were not measured over the follow-up, our analysis only includes levels measured at baseline. Consequently, we were not able to consider changes in hemoglobin levels and development or correction of anemia over the follow-up as additional time-varying exposures. Additionally, due to the observational nature of our study and despite adjustment for several relevant sociodemographic and clinical factors, we cannot exclude the possibility that the observed associations may be explained in part by residual confounding or reverse causality.

## Conclusions

In this prospective study among community-dwelling adults aged 70 years and older from five European countries, both prevalent anemia and lower hemoglobin levels at baseline were associated with higher odds of being at least pre-frail over three years. Our findings suggest the potential clinical relevance of hemoglobin levels in the context of pre-frailty and frailty and the importance of monitoring hemoglobin levels, even in generally healthy older adults.

## Data Availability

Data described in the manuscript, code book, and analytic code will not be made available to allow primary researchers of the DO-HEALTH Research Group to fully exploit the dataset. The data will be made available to external researchers in a second step according to a controlled access system.
